# Ultramicrosensors based on transition metal hexacyanoferrates for scanning electrochemical microscopy

**DOI:** 10.3762/bjnano.4.72

**Published:** 2013-10-14

**Authors:** Maria A Komkova, Angelika Holzinger, Andreas Hartmann, Alexei R Khokhlov, Christine Kranz, Arkady A Karyakin, Oleg G Voronin

**Affiliations:** 1 Faculty of Chemistry, M.V. Lomonosov Moscow State University, Moscow, Russia; 2 Institute of Analytical and Bioanalytical Chemistry, University of Ulm, Ulm, Germany; 3 Faculty of Physics, M.V. Lomonosov Moscow State University, Moscow, Russia

**Keywords:** energy related, hydrogen peroxide, nanomaterials, nickel hexacyanoferrate, Prussian Blue, scanning electrochemical microscopy, ultramicroelectrodes

## Abstract

We report here a way for improving the stability of ultramicroelectrodes (UME) based on hexacyanoferrate-modified metals for the detection of hydrogen peroxide. The most stable sensors were obtained by electrochemical deposition of six layers of hexacyanoferrates (HCF), more specifically, an alternating pattern of three layers of Prussian Blue and three layers of Ni–HCF. The microelectrodes modified with mixed layers were continuously monitored in 1 mM hydrogen peroxide and proved to be stable for more than 5 h under these conditions. The mixed layer microelectrodes exhibited a stability which is five times as high as the stability of conventional Prussian Blue-modified UMEs. The sensitivity of the mixed layer sensor was 0.32 A·M^−1^·cm^−2^, and the detection limit was 10 µM. The mixed layer-based UMEs were used as sensors in scanning electrochemical microscopy (SECM) experiments for imaging of hydrogen peroxide evolution.

## Introduction

The detection of hydrogen peroxide (H_2_O_2_) is of great importance in monitoring of food and the environment [1] as well as clinical [2], biological and chemical studies [3]. For example, hydrogen peroxide is a marker of inflammatory diseases [4]. Moreover, in fuel cells research, hydrogen peroxide is one of the key molecules as it is produced in the cathode chamber of the hydrogen–oxygen fuel cells causing degradation of the proton-exchange membranes [5]. Investigations of the local distribution of hydrogen peroxide on the surface of living cells and electrode materials as well as the in vivo analysis requires sensors with a size of 25 μm and less. For such electrodes (ultramicroelectrodes, UME) the thickness of the diffusion layer is comparable to the diameter of the electrode resulting in enhanced mass transport in comparison to macroscopic electrodes and thus leading to improved sensitivity and detection limits [6].

There are a number of studies which demonstrate the detection of H_2_O_2_ in SECM experiments based on its oxidation at bare platinum electrodes at a potential of 600 mV vs SCE [7–13]. However, such a high oxidation potential is often disadvantageous for real-world applications as interfering compounds may be co-oxidized. Prussian Blue (PB) is the most advantageous hydrogen peroxide transducer [14–16] due to its higher activity in H_2_O_2_ reduction and oxidation reactions, higher selectivity for hydrogen peroxide reduction in the presence of oxygen, and insensitivity to the presence of reducing compounds (e.g., ascorbate, paracetamol, etc.) [17]. We have already demonstrated miniaturized PB based electrodes with diameters ranging from 10 µm [18] to 125 µm [19]. For the electrodes with diameters of 125 µm, a record sensitivity of approximately 9 A·M^−1^·cm^−2^ in H_2_O_2_ detection was achieved. However, the stability of PB in neutral aqueous solutions is not sufficient in respect to the long-term continuous monitoring of high levels of H_2_O_2_ [20]. Moreover, different iron complexing agents (e.g., EDTA) are known to solubilize PB. This problem is particularly severe for PB-modified UME.

Operational stability of the PB can be improved by covering its surface with polymer films [21–22], by entrapment of the catalysts into sol–gel [23–25], and by conductive polymer matrixes [26–27]. In [20] we have demonstrated a novel approach for the stabilization of a sensor based on mixed iron-nickel hexacyanoferrates. Here, we report on the highly stable ultramicrosensors comprised of alternating films of iron and nickel hexacyanoferrates for the imaging of hydrogen peroxide distribution in SECM.

## Results and Discussion

Hexacyanoferrates were deposited onto UMEs with a diameter of 10 µm and 25 µm, respectively. Three types of UME were used: (i) carbon, (ii) platinum and (iii) platinum covered with an ion beam induced deposition (IBID)-generated Pt/C composite material [28]. Deposition of PB was carried out by using cyclic voltammetry (CV) as described elsewhere in detail [29]. PB was deposited using 5 cycles. A further increase of cycles led to a decreased stability. In spite of the good selectivity, PB-based electrodes showed low operational stability in batch measurements (see Table 1).

**Table 1:** Sensitivity and operational stability of the ultramicrosensors (pH 6, batch regime).

type of UME	type of film	sensitivity, mA/cm^2^	stability in 1 mM of H_2_O_2_, min

glassy carbon	PB	1600	10
glassy carbon	PB–Ni–HCF	81	240
platinum	PB	1050	15
platinum	PB–Ni–HCF	320	240
ion deposited Pt–C composite	PB	760	60
ion deposited Pt–C composite	PB–Ni–HCF	100	300

**Figure 1:**
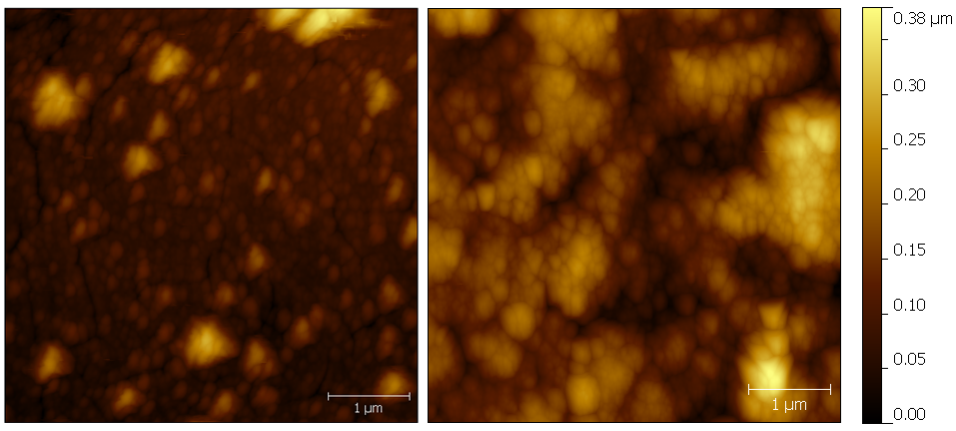
AFM topography images of Prussian blue layer (left) and Ni-HCF layer (right) deposited on top (contact mode images were recorded in air with 0.3 lines/s at a resolution of 512 × 512 lines/image).

Stabilized sensors were obtained by using a layer-by-layer deposition with mixed layers of PB and Ni–HCF. Ni^2+^ and Fe(CN)_6_^3−^ ions tend to form insoluble precipitate in pure aqueous solutions. Therefore an excessive amount of supporting electrolyte (0.5 М KCl) was used during the deposition of Ni–HCF films. Deposition of Ni–HCF was performed by using CV as described elsewhere [20]. Every two cycles of PB deposition were followed by 2 cycles of Ni–HCF deposition forming one “bilayer”. AFM images of Prussian blue and Ni–HCF deposited on top are shown in Figure 1. After the last deposition step the electrodes were activated by CV as described in the Experimental section of this manuscript.

**Figure 2:**
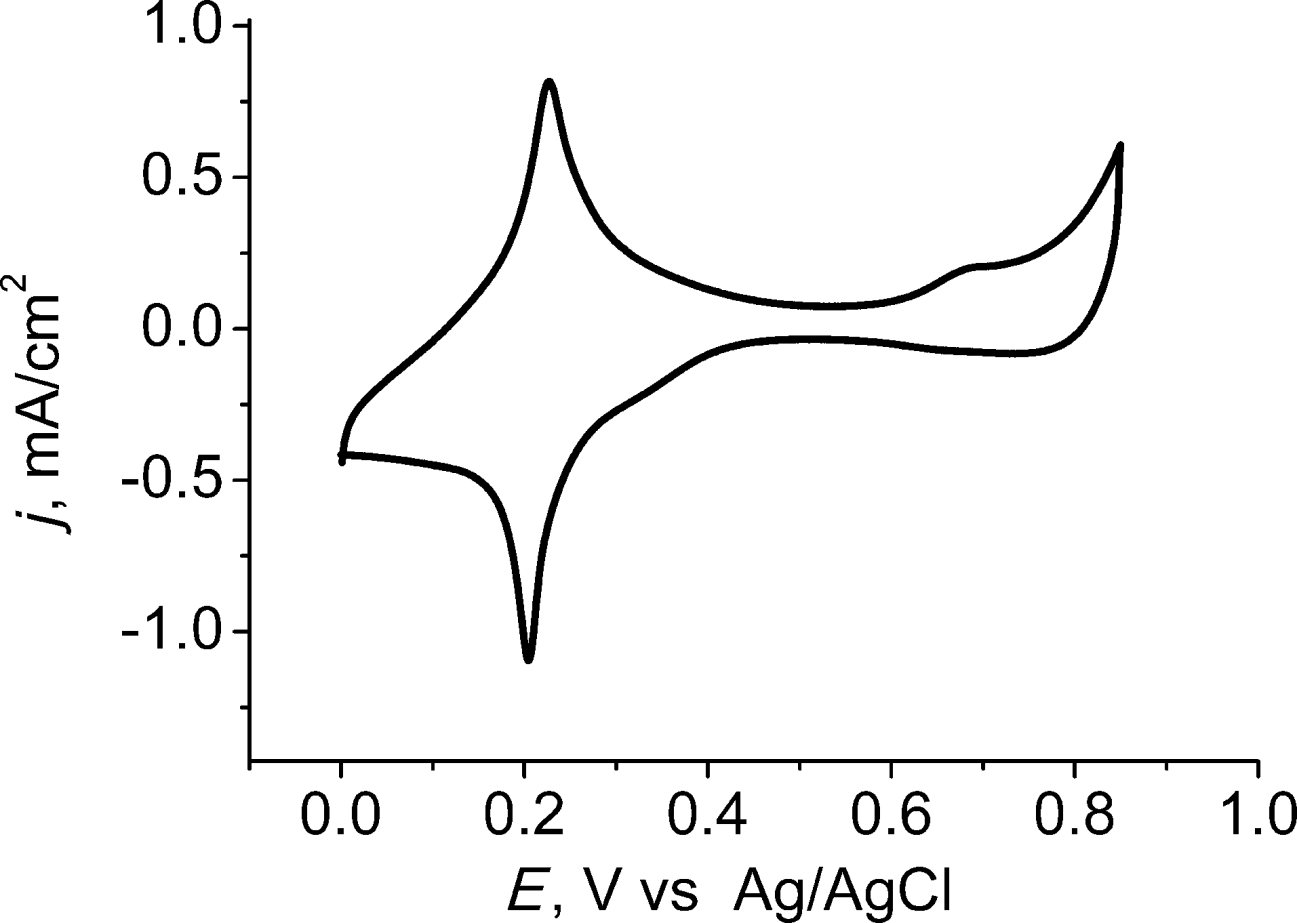
Cyclic voltammogram of three PB-Ni-HCF bilayers deposited on a platinum ultramictroelectrode (0.1 M KCl and 0.1 M HCl, sweep rate 20 mV·s^−1^).

Increasing the number of bilayers from one to three resulted in a higher sensitivity of the modified electrodes. A continued increase of the number of PB–Ni–HCF bilayers resulted in a loss of mechanical stability. Therefore, all further experiments were carried out with electrodes modified with three bilayers. A typical cyclic voltammogram of a 3-layer-modified microelectrode in supporting electrolyte solution is shown in Figure 2.

**Figure 3:**
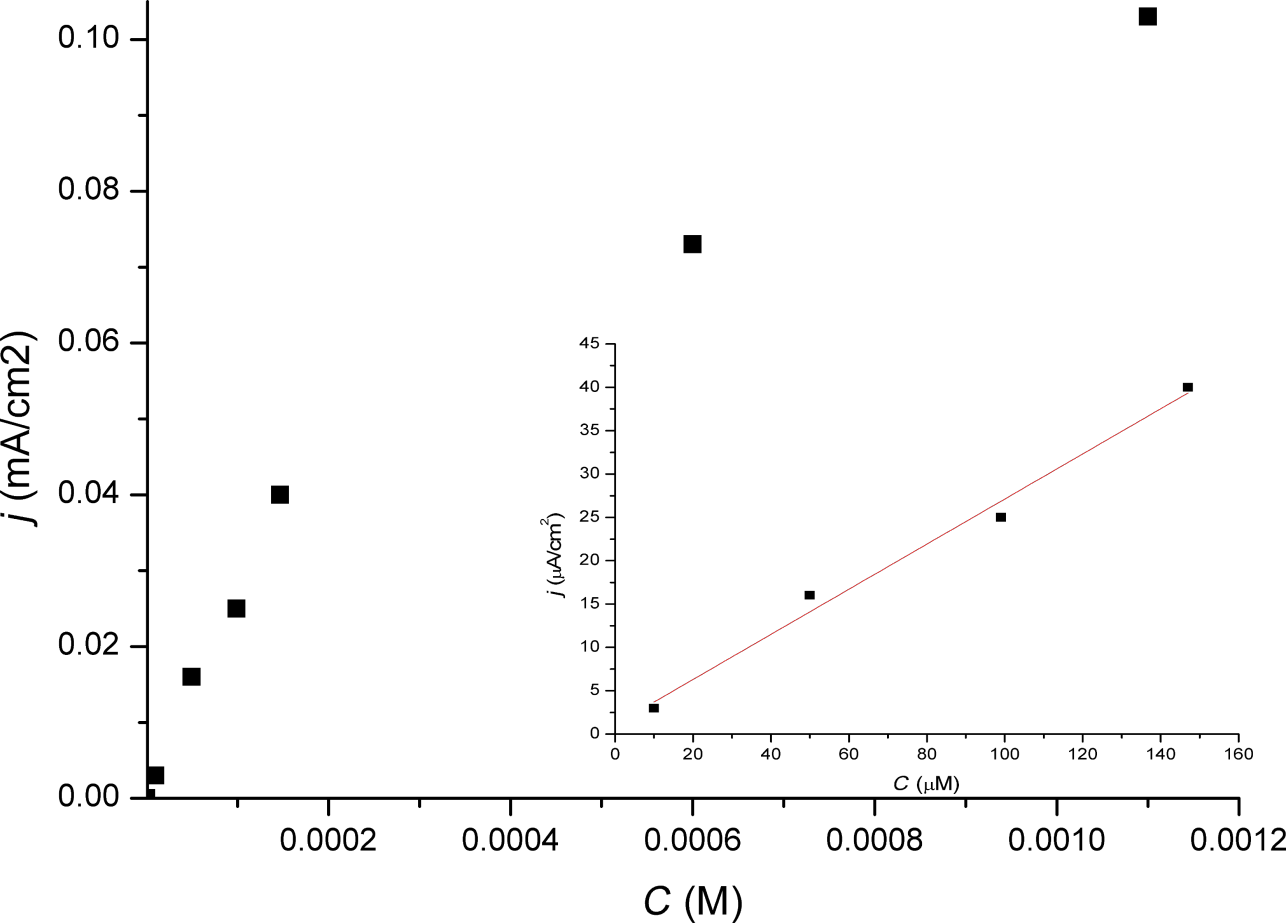
Standard addition curve for hydrogen peroxide recorded at 0 mV vs Ag/AgCl at a platinum UME (diam. of 25 µm) covered with three bilayers of PB–Ni–HCFs.

Table 1 summarizes the comparison of sensitivity and stability of PB–Ni–HCF-sensors using different electrode materials with data for UMEs only modified with PB. The measurements were carried out in a batch regime. The sensors with mixed layers showed a significantly improved stability and an expected decrease in sensitivity. Figure 3 shows an exemplary calibration curve for a microsensor based on PB–Ni–HCF mixed layers deposited on a platinum UME (diam. of 25 µm).

**Figure 4:**
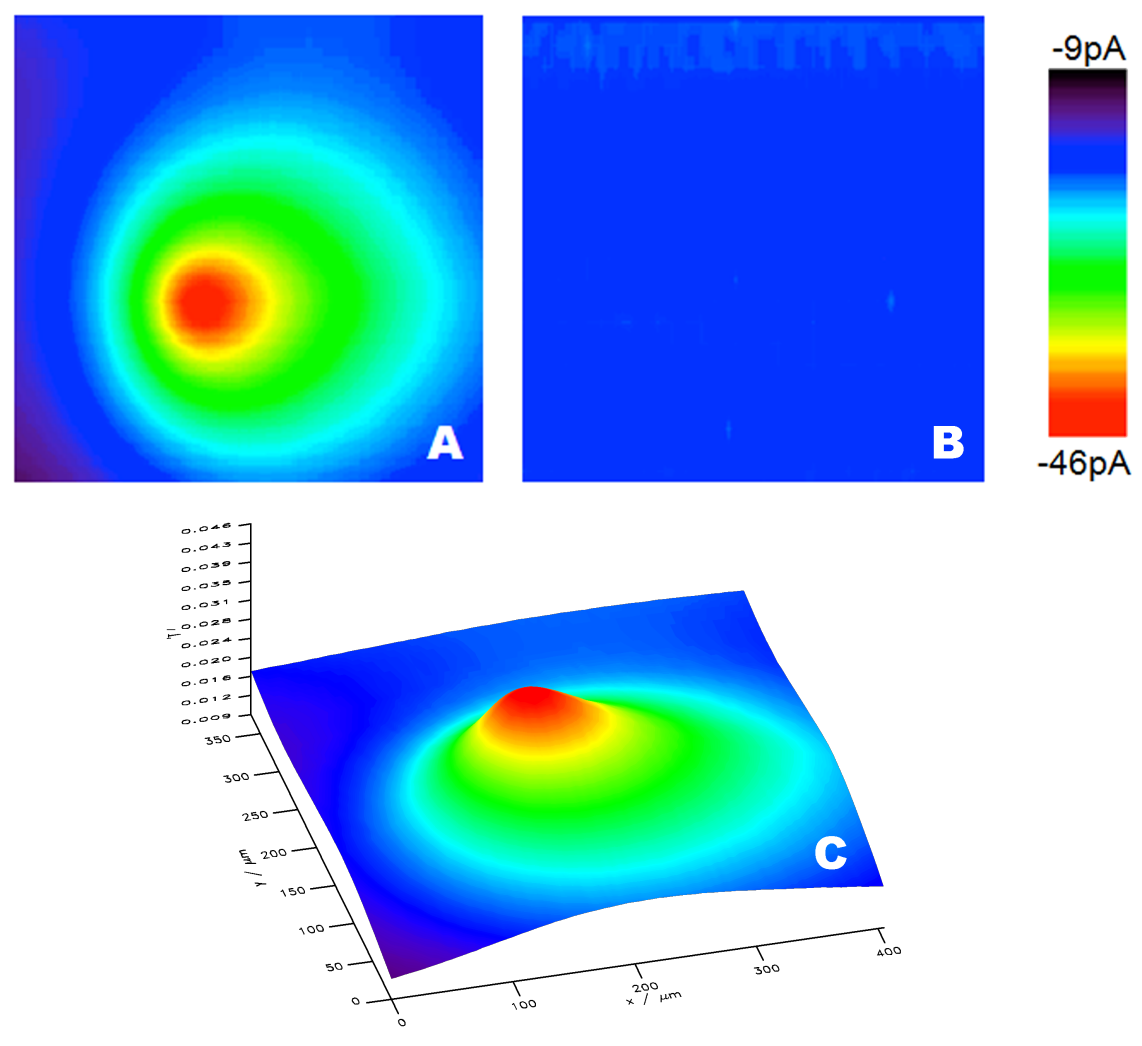
SECM image of an H_2_O_2_-generating gold electrode (diam. of 25 µm). A and B are 2D plots of images recorded with a platinum UME (diam. of 25 µm) covered with three layers of PB–Ni–HCF and with Ni–HCF, respectively. C illustrates a 3D plot of A.

PB–Ni–HCF-modified platinum microelectrodes were also applied in SECM experiments to map hydrogen peroxide profiles in substrate-generation-tip-collection mode (see Figure 4). As clearly visible in the SECM image, the reduction current significantly increased when the PB–Ni-modified electrode was scanned towards the center of the H_2_O_2_-generating gold electrode (Figure 4A). Control experiments were carried out by repeated SECM scans with three different scanning electrodes: (i) a blank platinum electrode (biased at 0 V vs Ag/AgCl), (ii) a platinum electrode modified only with Ni–HCF, and (iii) a PB–Ni–HCF-modified UME with no potential applied to the H_2_O_2_ generating gold electrode. In all control experiments a significant signal was not recorded. After the imaging experiments, the integrity of the film was confirmed by CV recorded in 0.1 M HCl/KCl.

## Conclusion

Ultramicrosensors for the detection of hydrogen peroxide with increased stability have been developed. It was shown that the electrodeposition of multiple PB–Ni–HCF bilayers on UME provides a significantly enhanced stability of the electrocatalytic films for different electrode materials. UMEs modified with PB–Ni–HCF films retained more than 95% of the initial catalytic activity during at least 5 hours continuous monitoring in 1 mM hydrogen peroxide.

## Experimental

Experiments were carried out in solutions prepared with Millipore water (resistivity 18.2 MΩ). All inorganic salts, organic solvents, and hydrogen peroxide (30% solution) were obtained at the highest purity from Sigma-Aldrich. Polishing materials were obtained from Leco Instruments GmbH and Allied High Tech Products Inc. Gold and platinum microwires were purchased from Goodfellow. The micro glassy carbon electrodes were obtained from ESA Biosciences Inc.

The electrochemical experiments were conducted in a three-electrode setup using either a CHI842B bipotentiostat or a μ-Autolab Type III (Eco Chemie) potentiostat as described in detail elsewhere [18]. An Ag/AgCl was used as a reference electrode and platinum was used as a counter electrode.

Microelectrodes were prepared as described elsewhere [30] by sealing platinum or gold wires (25 μm and 10 µm in diameter, respectively) under vacuum in borosilicate glass (Hilgenberg) or soda lime glass, respectively, followed by consecutive grinding, and polishing steps. Electrodes were then cleaned in an ultrasonic bath for 15 minutes. Circular Pt/C composite layers were deposited onto a microelectrode using a focused ion beam gas-assisted process (Quanta 3D FEG, FEI Eindhoven). The circular Pt/C composite were deposited on 10 µm Pt electrodes and had a radius of approx. 6.5 µm and a thickness of approx. 150 nm (ion beam current: 300 pA and a dwell time of 200 ns) with a ratio of carbon to platinum in the range of 60 atom % C to 24 atom % Pt [28]. Cyclic voltammetry, optical microscopy, and AFM (5500 AFM, Agilent Technologies) imaging were performed for characterizing the fabricated microelectrodes.

The deposition of PB was carried out as described in detail elsewhere [18]. The electrodeposition of nickel hexacyanoferrate (NiHCF) was carried out in a non-colloid solution containing 1 mM NiCl_2_ and 0.5 mM K_3_[Fe(CN)_6_] with an excessive amount of supporting electrolyte (0.1 M HCl and 0.5 M KCl), while cycling the electrode potential between 0 and 0.85 V at a scan rate of 100 mV/s applying 20 scans. After the deposition of NiHCF, the electrodes were rinsed with MilliQ water (Millipore MilliQ system) and and tempered at 80 °C for 0.5 h.

The deposition of the mixed films was performed as follows. First, a layer of PB was deposited by cyclic voltammetry as described above applying 2 scans. The electrodes were rinsed with distilled water and dried at 80 °C for 15 min. Then, the deposition of NiHCF was carried out by cyclic voltammetry applying 2 cycles. All layers (except the first and the last ones) were synthesized without temperature treatment. The activation was carried out by CV applying 10 cycles in 0.1 M KCl and 0.1 M HCl solution within the limits of 0.00 to +0.85 V at a scan rate of 40 mV/s and was performed after electrosynthesis of the last layer. Then the electrodes were dried at 80 °C for 0.5 h.

SECM measurements were performed in generation–collection mode as described in detail elsewhere [18]. A gold microelectrode (25 μm in diameter) was used for generating hydrogen peroxide (bias: −0.4 V vs Ag/AgCl). Platinum microelectrodes covered with PB, PB/NiHCF and NiHCF were used to detect the reduction current of hydrogen peroxide at 0.00 V vs Ag/AgCl. The microelectrode modified by metal cyanoferrate was positioned in close proximity to the hydrogen peroxide generating UME in feedback mode recording the Faraday current of the Au microelectrode during the approach of the modified electrode. Prior to the approach curve, the electrodes were positioned centered to each other using an optical microscope. The non-biased modified UME was then approached to the Au microelectrode while the feedback current at the Au UME was recorded in 10 mM ferrocyanide/0.1 M KCl. A negative feedback signal was obtained due to the hindered diffusion of ferrocyanide towards the Au UME when the modified electrode is in the vicinity. The SECM image was then recorded in constant-height mode at a distance of 50 µm (determined by Mira software package, G. Wittstock, University of Oldenburg).
